# Assessment of clinical and logistical contribution in a Norwegian helicopter emergency medical service using integrated data: a retrospective observational study

**DOI:** 10.1186/s13049-026-01615-3

**Published:** 2026-04-17

**Authors:** Lars E. Næss, Oddvar Uleberg, Andreas Asheim, Andreas Krüger, Eivinn Skjærseth, Ole Erik Ulvin, Jostein Dale, Rune Sætre, Helge Haugland

**Affiliations:** 1 Department of Research and Development, The Norwegian Air Ambulance Foundation, Oslo, Norway; 2 Department of Emergency Medicine and Prehospital Services, St. Olav’s University Hospital, Trondheim, Norway; 3 Department of Circulation and Medical Imaging, Norwegian University of Science and Technology, Trondheim, Norway; 4 Centre for Health Care Improvement, St. Olav’s University Hospital, Trondheim, Norway; 5 Department of Clinical and Molecular Medicine, Norwegian University of Science and Technology, Trondheim, Norway; 6 Department of Computer and Information Science, Norwegian University of Science and Technology, Trondheim, Norway

**Keywords:** Emergency Medical Services, Air Ambulances, Quality Indicators, Health Care, Patient Outcome Assessment, Emergency Medical Service Communication Systems, Electronic Health Records, Health Information Management, Health Data Administration, Information Systems

## Abstract

**Background:**

Physician‑staffed Helicopter Emergency Medical Services (HEMS) provide advanced pre‑hospital care, rapid transfer to hospital, and access to remote areas. These services are costly with limited capacity; therefore, their value depends on meaningful patient benefit. Although clinical and logistical capabilities are essential for selected patient groups, no unified measure of service benefit exists. In 2017 an international expert panel proposed a set of quality indicators (QIs) for Physician‑Staffed Emergency Medical Services (P‑EMS). Since 2021 the Trondheim HEMS has used these QIs to assess clinical and logistical contribution in completed missions. This study aims to describe these contribution assessments and relate them with descriptive data and established severity measures from integrated Emergency Medical Services (EMS) and hospital data.

**Methods:**

Physician‑reported assessments of clinical and logistical contribution from Trondheim HEMS (2022–2024) were linked with data from the Emergency Medical Communication Centre, HEMS records and hospital records. Contribution assessments and mission characteristics were examined using descriptive statistics. Associations and convergence between contribution assessments, severity measures and patient characteristics were explored using multivariable regression models.

**Results:**

HEMS contribution was assessed for 2,286 missions. Of these, 1,696 (74%) were judged as beneficial, including 1,173 (51%) with logistical contribution and 897 (39%) with clinical contribution, with an overlap of 374 missions (16%) showing both. Logistical contribution was associated with conditions requiring rapid transfer to definitive treatment, while clinical contribution was associated with potentially critical diagnoses, higher severity scores, higher mortality rates, and greater hospital utilisation. Retrospectively, 590 missions (26%) were classified as having no contribution, more often involving younger patients and potentially critical but uncertain conditions.

**Conclusions:**

Across three years of retrospective physician‑reported assessments, three quarters of HEMS missions were retrospectively considered beneficial, reflecting approximately 50 percent logistical contribution and 40 percent clinical contribution, with a 16 percent overlap. The remaining 26 percent were viewed as not beneficial. Assessments of relative contributions varied between physicians suggests differing interpretations of the criteria, highlighting the need to strengthen a shared understanding of the underlying concepts.

**Supplementary Information:**

The online version contains supplementary material available at 10.1186/s13049-026-01615-3.

## Introduction

### Background

Physician staffed Helicopter Emergency Medical Services (HEMS) provide advanced pre-hospital care, rapid long-distance transport, and access to patients in areas inaccessible by road [1, 2]. These capabilities may be crucial in critical or time-sensitive conditions [3–5], and HEMS involvement has been associated with improved outcomes in severe trauma, traumatic brain injury, paediatric trauma, acute stroke, cardiac emergencies and respiratory failure [6–11]. However, evidence of HEMS benefit remains uncertain in other conditions [4, 12], and the cost-effectiveness of the service is debated [13–16].

Efficient HEMS dispatch requires a careful balance between undertriage and overtriage [17]. Overtriage is primarily a system level concern, but it may also affect subsequent patients by tying up scarce resources. By contrast, undertriage directly affects patient outcomes [14]. Because actual need often becomes evident only in retrospect, pre-hospital triage involves inherent uncertainty [18]. Even with all relevant data available, it is difficult to measure the benefit of HEMS against a theoretical counterfactual course, and no universally accepted benefit measure exists despite several proposals [19–21]. To address this, systematic quality evaluations have been recommended [22, 23]. In 2017, the EQUIPE consensus panel (Establishing Quality Indicators in Physician-staffed Emergency Medical Services) proposed quality indicators (QIs) for physician-staffed emergency medical services (P-EMS) [24], which were later tested and benchmarked in a multicentre study [25].

Since late 2021, the physician-staffed Trondheim HEMS has routinely recorded physician-reported process indicators during mission follow-up using a dedicated QI registration application. Two indicators capture aspects of the contribution of physician-staffed HEMS: QI 12, provision of advanced medical treatment (clinical contribution), relating to the capabilities of the physician and other crewmembers; and QI 13, provision of significant logistical support (logistical contribution), relating to helicopter capabilities. These measures have been used as proxies for beneficial HEMS missions in local research [26, 27], but remain unvalidated. Medical emergencies involve complex care pathways that require integrated data for analysis [11, 28]. Over the past decade, Trondheim HEMS has established information technology (IT) infrastructure to link routinely collected HEMS data with Emergency Medical Communication Centre (EMCC) data and hospital records, enabling comprehensive evaluation of HEMS trajectories.

### Objectives

The aim of this study is to describe physician‑reported EQUIPE QI scores for contribution (QI 12 and QI 13) from Trondheim HEMS, and to explore their convergence with operational measures, severity measures, and patient outcomes across linked HEMS, EMCC and hospital data.

## Methods

### Study design

We conducted a retrospective observational study linking Trondheim HEMS contribution assessments from 1 January 2022 to 31 December 2024 with routinely collected EMS data (including HEMS), and hospital records.

### Setting

**Fig. 1:**
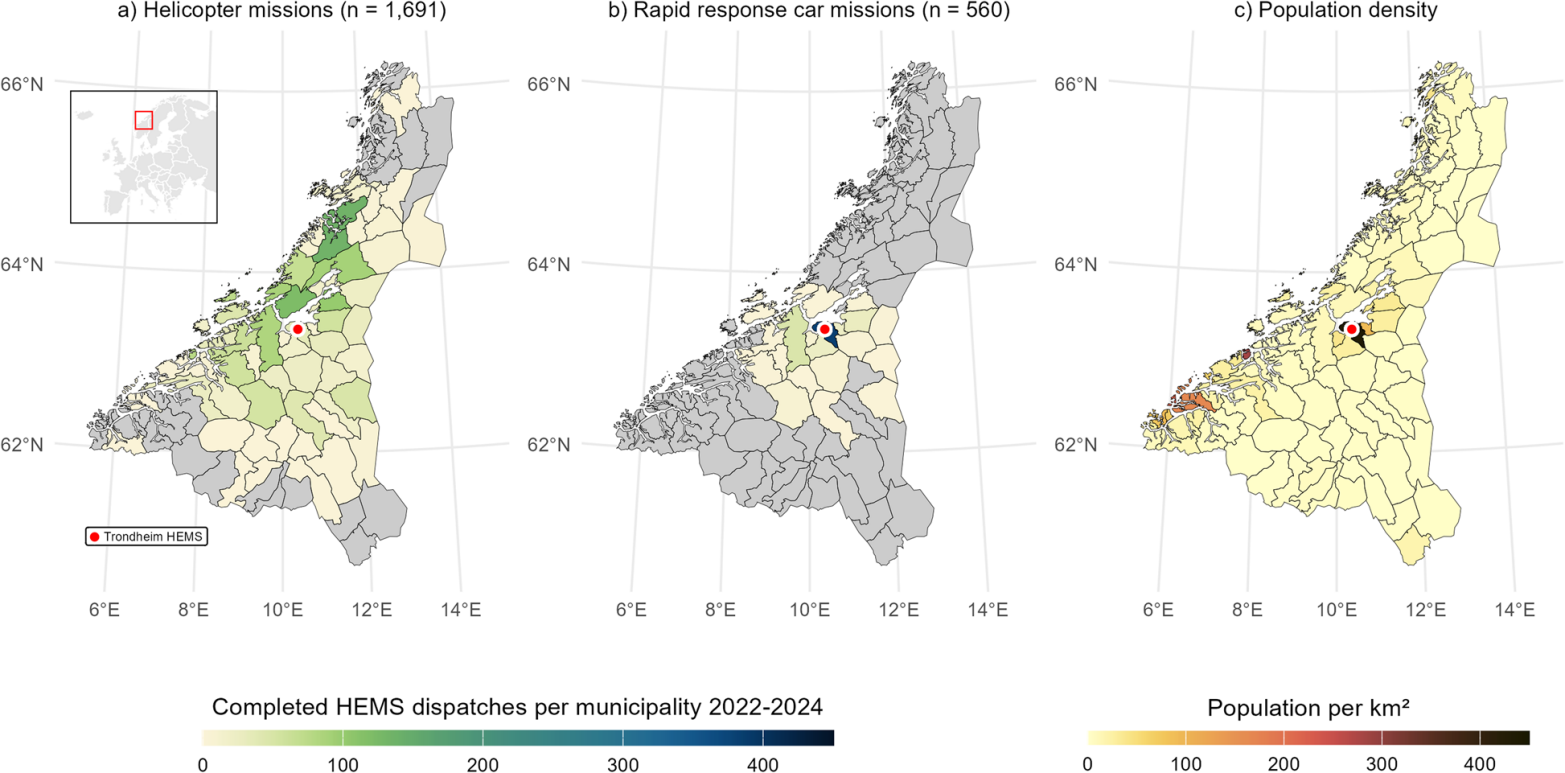
Trondheim HEMS service area. **a** Number of completed helicopter missions per municipality. **b** Number of completed rapid response car mission per municipality. **c** Population density per municipality 2024 [34]. HEMS – Helicopter Emergency Medical Services

The Central Norway Regional Health Authority (Helse Midt RHF) covers Trøndelag and Møre og Romsdal counties, spanning 56,559 km^2^ and serving 753,600 inhabitants (2024). The region comprises sparsely populated mountainous and coastal areas and urban centres surrounding the main cities [29]. Trondheim, the regional capital, has 214,565 inhabitants (2025) [30]. St. Olav’s University Hospital, located in Trondheim, is the tertiary referral hospital and trauma centre for the region [31]. Trondheim HEMS is one of three regional physician staffed HEMS units, responding to approximately 1,200 requests annually. The HEMS crew consists of a board-certified anaesthesiologist, a crew member (nurse or paramedic), and a pilot. Eight consultant anaesthesiologists from St. Olav’s University Hospital rotate as attending physicians. Trondheim HEMS mainly responds by helicopter, however in incidents closer to the HEMS base, or when adverse weather prevents helicopter dispatch, the HEMS physician and crew member responds by a rapid response car (RRC), supported by other ground-based EMS resources. The HEMS physician also assists with inter‑hospital ambulance transfers of intensive care patients. Missions are categorized as primary (scene response), secondary (inter-hospital transfers), or search and rescue (SAR). The service area and distribution of deployed primary assets are shown in Fig. 1a and b, along with population density in Fig. 1c. HEMS dispatches are initiated by the local EMCC based on national guidelines [32]. For each request, the local EMCC operator contacts a dedicated regional HEMS coordinator, who assesses the operational feasibility and activates the most appropriate HEMS unit. The regional HEMS coordinator has specific training in aeromedical operations, flight safety (including flight following), and resource optimisation. The coordinator is responsible for flight‑operative decisions and allocation of regional HEMS resources, while the local EMCC retains responsibility for medical triage and patient‑care follow-up. This two‑level structure ensures that dispatch decision-making is integrated between medical and flight‑operative expertise [33]. On request, the on-call HEMS physician makes the final dispatch decision based on medical need, while the pilot assesses operational readiness, including weather [26].

### Data selection and study cohort

**Fig. 2:**
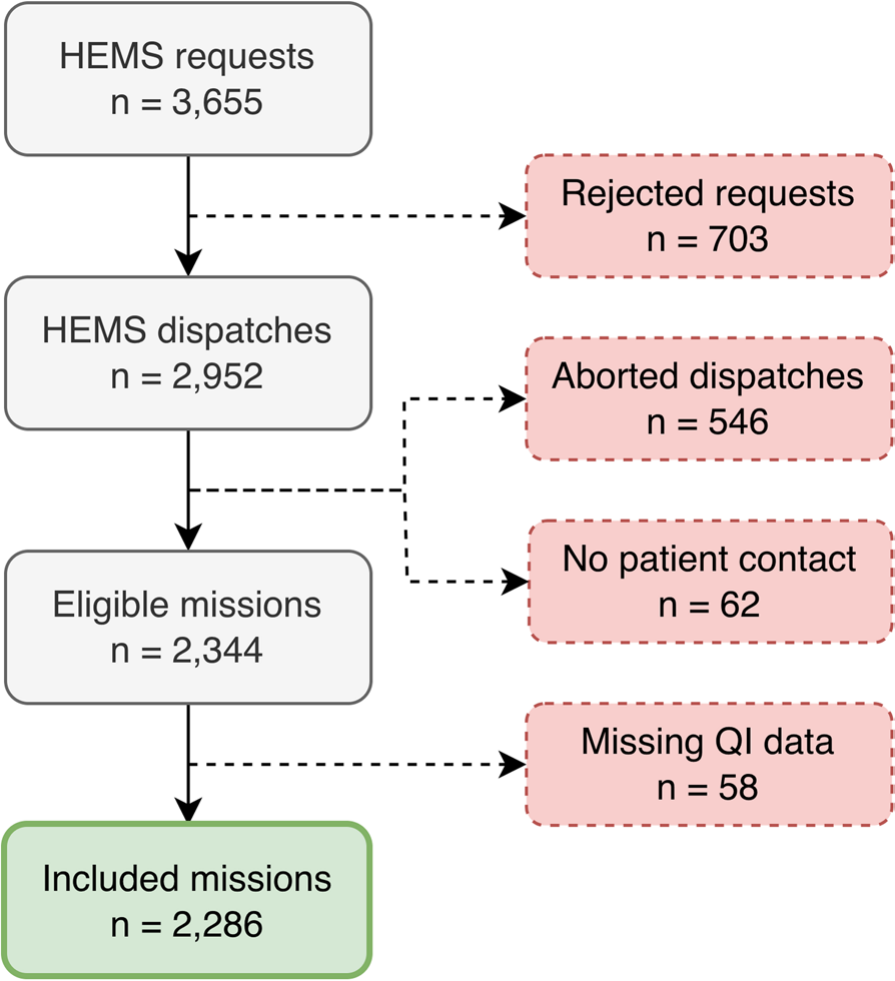
Flowchart for selection of eligible study data. HEMS – Helicopter Emergency Medical Services. QI – Quality Indicator

Based on all requests for Trondheim HEMS registered by the local EMCC from 1 January 2022 to 31 December 2024, we included dispatches that resulted in patient contact with documented assessment of contribution (EQUIPE QI 12 and/or QI 13). We excluded requests that were rejected, aborted, or without patient contact. Missions completed without QI assessment were also excluded. Recorded reasons for rejection or abortion were: no medical need, patient not fit for transport, patient declared dead upon arrival on scene, technical problems, weather conditions, duty time limits, or concurrency conflicts [35]. Figure 2 presents the study selection process. For incidents with duplicate EMCC entries, such as a rejected helicopter mission followed by an accepted RRC dispatch, we retained the final accepted entry with patient contact. For dispatches involving several patients, one primary patient was selected based on transport status, severity score, and the highest overall contribution score.

### Data sources

Incident data were retrieved from the EMCC system AMIS (Omda AS, Oslo) and the HEMS documentation system LABAS (Normann IT, Trondheim). Self-reported QI-data were collected in a dedicated QI application (Helse-Midt IT AS, Trondheim). Hospital care data were retrieved from the Nimes administrative system at St. Olav’s University Hospital (LOGEX AS, Oslo). Demographic information (sex, date of birth, and date of death) was retrieved from the Norwegian National Population Register via the local Patient Administrative System.

### Data access and handling

Data were accessed via the data warehouse of the Central Norway Regional Health Authority (HMN Datavarehus, Hemit HF, Trondheim), a SQL (Structured Query Language) Server–based platform with controlled access. Multi-source SQL data were extracted and imported into RStudio 2025.09.0 + 387 (Posit Software PBC, Boston) using ODBC and DBI packages [36, 37]. Data handling and analysis were conducted in RStudio using R version 4.5.1 (R Core Team, Vienna, 2025).

Incident data from EMCC and HEMS, along with QI data, were merged using an EMCC incident identification number. As incidents could involve multiple patients, HEMS patient records were linked to EMCC data by a combination of incident identification number and date of birth. A pseudo-anonymised patient identifier (PID) from HMN Datavarehus was used to link EMCC data with Hospital records, matched by PID and overlapping incident date-time intervals. Acute care pathway frameworks were adapted from the EMS Outcomes Project and European Emergency Data project [11, 28], with interactions illustrated in Supplementary Fig. 1.

SQL code for extracting and merging study data from multiple sources in the HMN Datavarehus SQL Server is available on GitHub: https://github.com/larsnaess/HEMS-contribution.

### Main measures

HEMS contribution was assessed using two contribution‑specific physician‑reported QIs developed by the EQUIPE panel for P‑EMS, QI 12 Advanced treatment (clinical contribution) and QI 13 Logistics (logistical contribution) [24]. Both indicators were recorded as binary responses ("yes" or "no"), with supplementary information provided for all "yes" responses. Missions with "no" response recorded for both indicators were classified as having "no contribution". In our service, logistical contribution depends on helicopter capabilities and is therefore only relevant for helicopter dispatches.

Below is an outline of the criteria for responses for each indicator based on the assessment tool (QI application). Multiple answers were allowed. Screen‑shots from the QI application are provided as Supplementary Figs. 3 and 4 (Norwegian language). The original criteria from the EQUIPE framework are available in the supplementary material of Haugland et al. [24].

#### “Clinical contribution”—QI 12 advanced treatment

Did HEMS perform advanced medical procedures on this mission?

*Yes*

- *Procedures offered only by HEMS*
- *Procedures also available from other prehospital resources than HEMS, but none of these were present at the scene*
- *Decision to refrain from unethical or unnecessary treatment*
- *Presence in particularly demanding situations*

*No*

The category “procedures also available from other prehospital resources, but none were present at the scene” is inherently context dependent. It may encompass a wide range of interventions, from procedures performed when HEMS is the first unit to arrive (such as CPR), to basic interventions delivered in remote areas where alternative providers cannot reach the patient in time, for example analgesia and fracture management in mountainous terrain.

The criteria “decision to refrain from unethical or unnecessary treatment” and “presence in particularly demanding situations” reflect more subjective elements of clinical judgement. Their interpretation depends on both the clinical and operational context, as well as the attending physician’s experience and approach. HEMS physicians are trained to manage complex and challenging situations involving severe illness, injury or death, and their presence may be essential to support patients, relatives, bystanders or other healthcare personnel at the scene. Their seniority and professional authority may also be required when declaring a patient deceased or when refraining from treatment that would be inappropriate or ethically unjustifiable.

#### “Logistical contribution” – QI 13 logistics

Did HEMS provide a logistical contribution with significant benefit to the patient compared with other resources?

*Yes*

- *Reduced transport time by* ≥ *30 min for time-critical conditions*
- *Reduced transport time by 15–29 min for time‑critical conditions*
- *Transport of patients from a scene not accessible to an ambulance*

*No*

### Other measures

HEMS severity assessments were classified according to the National Advisory Committee for Aeronautics (NACA) [38], Supplementary Table 1.

We used a set of fifteen procedures recorded in the HEMS journal and associated with serious illness or injury as an indicator of patient severity.

These were: cardiopulmonary resuscitation (CPR); defibrillation or electrical cardioversion; extracorporeal membrane oxygenation (ECMO); external pacing; mechanical chest compression device; intubation or tracheostomy; mechanical ventilation; central venous catheter (CVC) placement; general anaesthesia; incubator use; invasive monitoring; thoracic drain insertion; thrombolysis; and ultrasound examination.

Most of these procedures are exclusive to HEMS, although some may also be provided by other healthcare providers, such as CPR, defibrillation, and thrombolysis. As QI 12 encompasses both HEMS‑exclusive and non‑exclusive procedures depending on the circumstances, this list cannot be used as a direct reference standard, although an overall convergence between the assessment of clinical contribution and the provision of these procedures is expected.

The tentative HEMS diagnosis was coded using ICD-10 (International Classification of Diseases, 10th Revision) [39], and shortened to three characters to create broader diagnostic groups (e.g., both I21.0 and I21.9 shortened to I21).

Mortality was assessed as 30-day mortality from time of incident. Hospital stays were calculated as number of days admitted. To measure health service utilisation, we used diagnosis related group (DRG) points (obtained from hospital reimbursement data), as a proxy for hospital costs [40–42]. One DRG point was valued at NOK 50,252 [43], equivalent to EUR 4,396 based on the 2023 mid-year exchange rate of 11.4305 [44].

### Statistical analysis

Operational data, patient descriptors, and contribution assessments were summarised as counts and proportions. Percentages referred to all included missions unless otherwise stated. Continuous variables were summarised as medians with interquartile range (IQR, 25th to 75th percentile).

Associations between covariates and contribution assessments were examined using separate binary logistic regression models for logistical contribution, clinical contribution, and no contribution. These outcomes were analysed in separate models because the categories were not mutually exclusive. Covariates were drawn from EMCC data (sex, age, timestamps, and incident type). Incident type was classified according to the Norwegian Index for Medical Emergency Assistance (NIMN) [45]. We also included the identity of the attending physician from HEMS data as a covariate to adjust for rater variation.

Adjusted predicted probabilities with 95% confidence intervals (CI) were obtained using the “marginaleffects package” in R [46]. Age was modelled using natural splines (4 degrees of freedom) to allow for non-linear associations [47], with continuous covariates held at their means and categorical covariates at reference levels.

Associations between contribution assessments and hospital outcomes were analysed using logistic or linear regression, depending on the outcome scale.

R code for statistical analyses and for generating tables and figures is available on GitHub: https://github.com/larsnaess/HEMS-contribution.

## Results

Between 2022 and 2024, Trondheim HEMS received 3,655 requests, resulting in 2,344 registered patient contacts. Of these contacts, 2,286 were included in the analysis based on completed contribution assessments of HEMS contribution in the QI application (Fig. 2). Of the missions included, 80% (1,840) were primary, and the helicopter was dispatched in 74% (1,691). In the cohort, 64% (1,470) of patients were male, the median age was 61 years (IQR 35 to 74), and 86% (1,977) were admitted to hospital. Within 30 days, 20% (462) of patients died, about half of whom (234) were registered as NACA 7, meaning they were assessed and declared dead at the scene by HEMS (Table 1).

**Table 1:** Basic operational data and patient descriptors for missions included in the study

Descriptor	All dispatches	Helicopter dispatches	RRC dispatches	Other dispatches
Overall (%)	2286	1691 (74)	560 (24)	35 (2)
Mission type (%)				
Primary mission	1840 (80)	1323 (78)	510 (91)	7 (20)
Secondary mission	434 (19)	357 (21)	50 (9)	27 (77)
SAR/Other	12 (1)	11 (1)	< 5 (-)	< 5 (-)
Sex (%)				
Female	816 (36)	604 (36)	193 (34)	19 (54)
Male	1470 (64)	1087 (64)	367 (66)	16 (46)
Patient age^*^ (%)				
0–17 years	323 (14)	227 (13)	94 (17)	< 5 (-)
18–49 years	473 (21)	326 (19)	141 (25)	6 (17)
50–66 years	560 (24)	443 (26)	113 (20)	< 5 (-)
67–79 years	696 (30)	520 (31)	160 (29)	16 (46)
80–89 years	210 (9)	159 (9)	45 (8)	6 (17)
Over 90 years	19 (1)	11 (1)	7 (1)	< 5 (-)
NACA score^*^ (%)				
NACA 1–3	487 (21)	397 (23)	89 (16)	< 5 (-)
NACA 4	859 (38)	727 (43)	128 (23)	< 5 (-)
NACA 5	456 (20)	322 (19)	109 (19)	25 (71)
NACA 6	248 (11)	123 (7)	123 (22)	< 5 (-)
NACA 7	234 (10)	120 (7)	111 (20)	< 5 (-)
HEMS Outcome (%)				
Hospital admission	1977 (86)	1511 (89)	435 (78)	31 (89)
No hospital admission	309 (14)	180 (11)	125 (22)	< 5 (-)
Mortality (%)				
Dead within 30 days	462 (20)	248 (15)	200 (36)	14 (40)
Alive after 30 days	1720 (75)	1354 (80)	346 (62)	20 (57)
Unknown 30-day mortality	104 (5)	89 (5)	14 (3)	< 5 (-)

^*^Unknown: age (*n* = 5), NACA (*n* = 2)

HEMS - Helicopter Emergency Medical Services, NACA - National Advisory Committee for Aeronautics

**Fig. 3:**
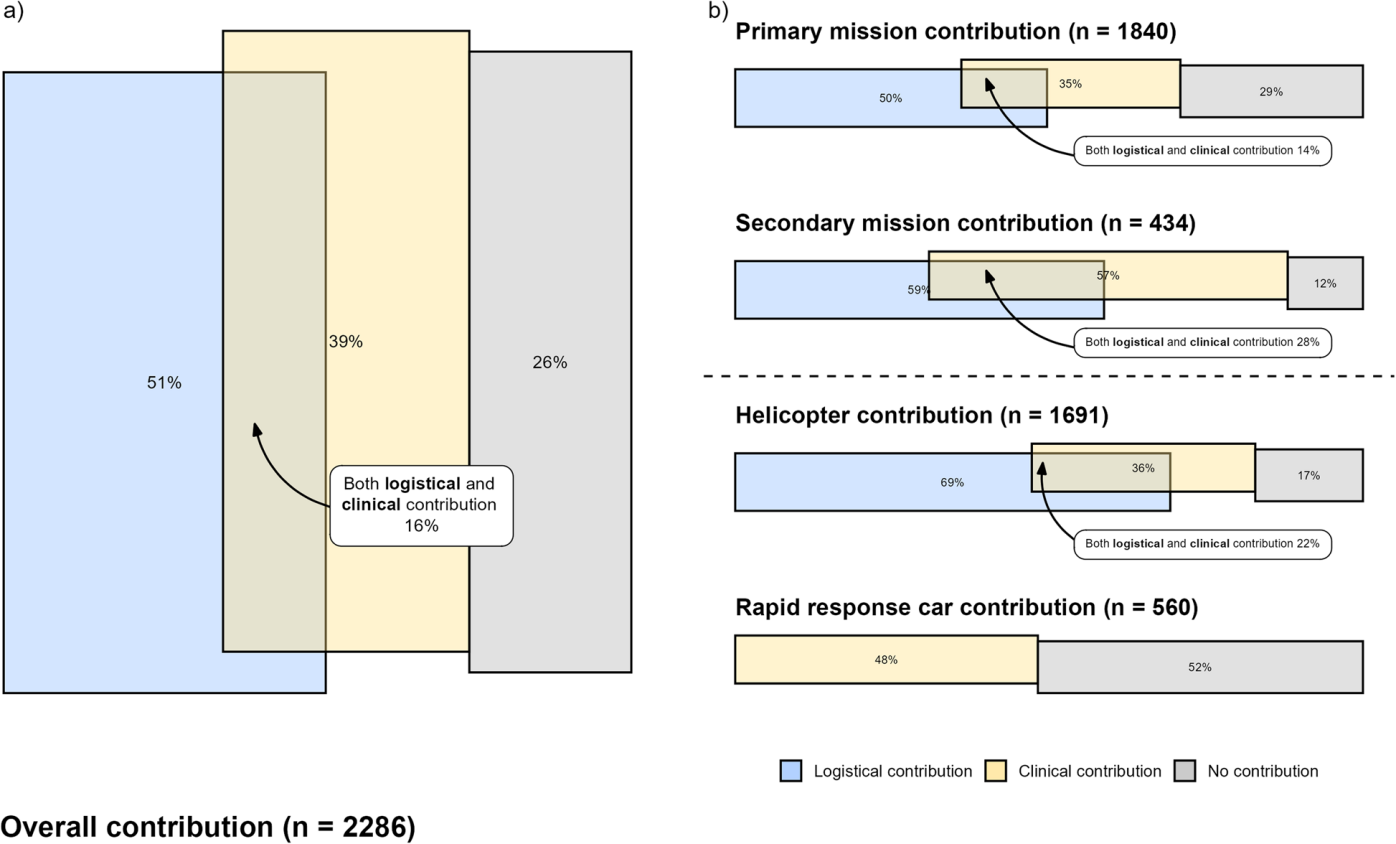
Contribution assessment according to EQUIPE quality indicators for (**a**) All eligible HEMS missions (n = 2,286) and (**b**) Eligible HEMS missions stratified by type and HEMS vessel. Search and Rescue (SAR) missions and incidents managed by other modalities were excluded due to low volume (n = 12). HEMS – Helicopter Emergency Medical Services

In total, 74% (1,696) of missions were judged beneficial. Of these 51% (1,173) had logistical contribution and 39% (897) had clinical contribution, 16% (374) had both. Thus 26% (590) were classified as no contribution. The distribution is shown in Fig. 3a. In sub-analyses, logistical contribution (1,173) was predominantly time savings, 92% (1,077). Clinical contribution (897) mainly comprised HEMS exclusive procedures, 88% (791), and standard EMS procedures delivered when no other provider was present, 8% (69) (Supplementary Fig. 2). Contribution assessment varied between physicians. Median proportions were 53% (IQR 47%–55%) for logistical contribution, 39% (IQR 29%–50%) for clinical contribution, and 26% (IQR 21%–31%) for no contribution.

Secondary missions had higher proportions of both logistical and clinical contribution, 59% (255) and 57% (248), than primary missions, 50% (914) and 35% (642). Thus, no contribution was more frequent in primary missions, 29% (535), compared to secondary missions, 12% (52), (Fig. 3b). Of 1,691 helicopter missions 69% (1,172) involved logistical contribution. RRC missions showed higher proportions of clinical contribution, 48% (270), and no contribution, 52% (290), than helicopter missions, 36% (602) and 17% (290), respectively (Fig. 3b). Further details are provided in Supplementary Table 2.

**Fig. 4:**
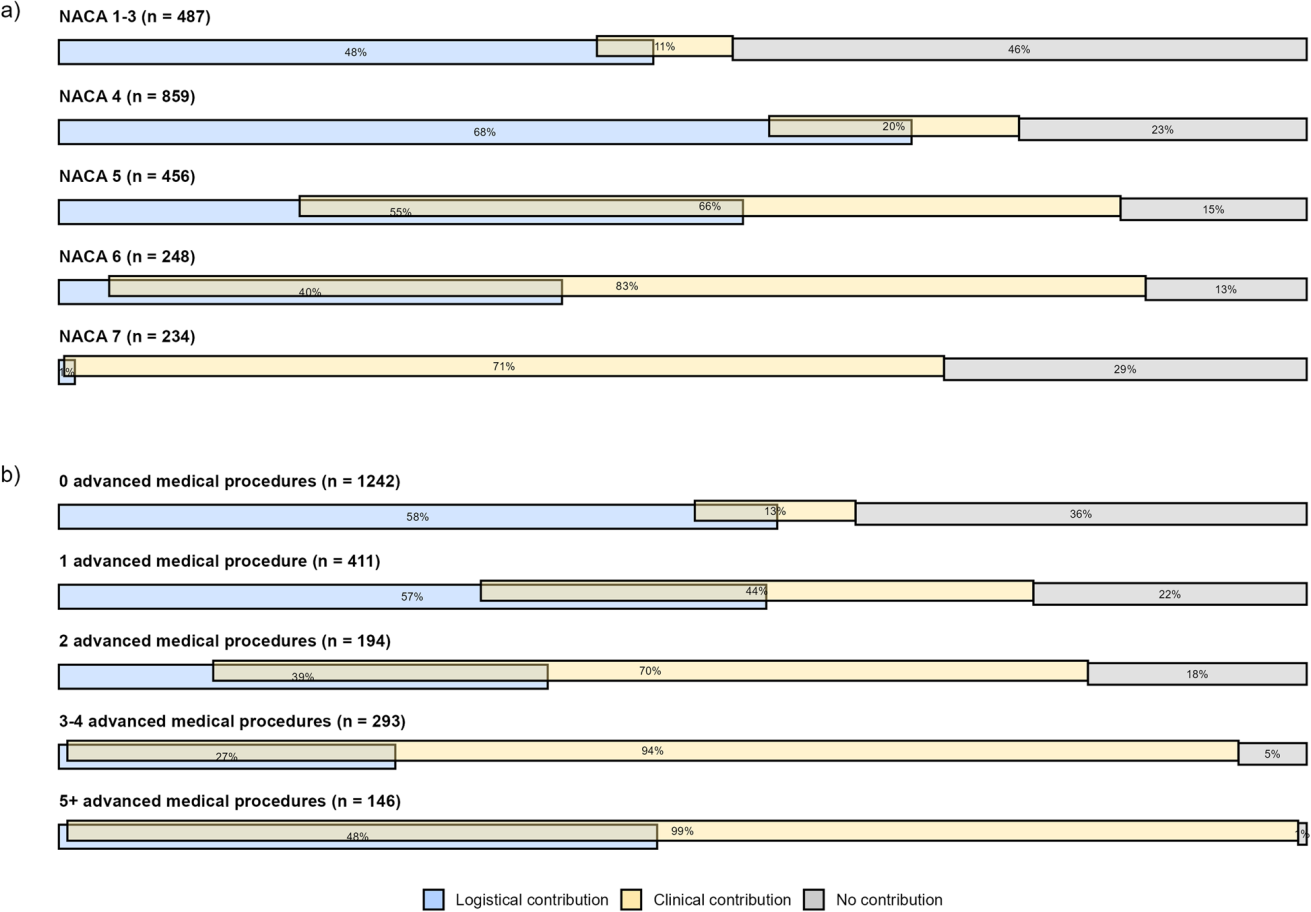
Associations between contribution and (**a**) NACA score (severity assessment), **b** Number of advanced medical HEMS procedures. HEMS – Helicopter Emergency Medical Services, NACA – National Advisory Committee for Aeronautics

Logistical contribution was highest in NACA 4, 68% (587) and declined with increasing severity. Clinical contribution rose from 11% (53) in NACA 1–3 to 83% (206) in NACA 6, then fell to 71% (165) in NACA 7 (Fig. 4a).

Logistical contribution decreased as procedure count increased, reaching a low of 27% (79) for 3–4 procedures, then rising to 48% (70) at five or more procedures. Clinical contribution increased with procedure count (Fig. 4b). Further details are provided in Supplementary Table 2.

Among 274 different ICD‑10 diagnosis codes recorded by HEMS, eighteen codes accounted for 60% (1,368) of cases, each occurred at least twenty times (Supplementary Table 3). Within these frequent codes, logistical contribution was highest for myocardial infarction (I22 97%, I21 95%) and stroke (I63 87%, I64 87%). Clinical contribution was highest for patient recorded as cardiac arrest (I46, 77%), aortic aneurysm or dissection (I71, 72%) and intracerebral haemorrhage (I61, 62%). No contribution was most common for syncope/collapse (R55, 68%) and convulsions (R56, 64%).

**Fig. 5:**
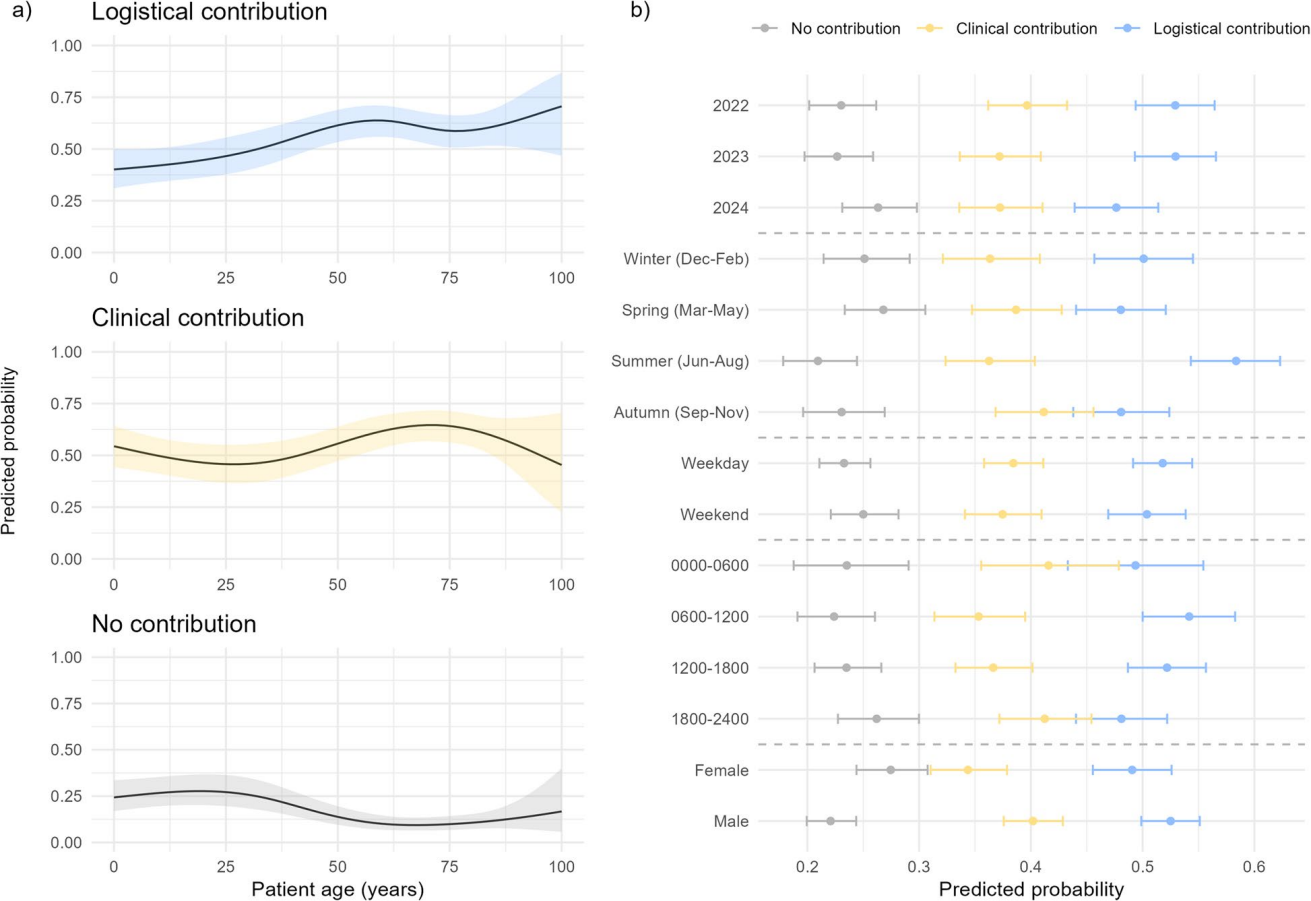
Predicted probability of contribution with 95% confidence intervals. Covariates: age, sex, year, season, weekday, working hours, physician. **a** Estimated contribution by patient age, modelled using natural splines with four degrees of freedom. **b** Estimated contribution across time factors and patient sex

Using pre-known variables recorded at dispatch, adjusted model-based predicted probabilities showed clear age patterns. Logistical contribution increased with age. Clinical contribution also increased with age but plateaued above 75 years (any decline at the oldest ages was small and within the confidence interval). No contribution was highest in the youngest, then decreased with age, with a slight increase in the oldest group (Fig. 5a).

The predicted probability of logistical contribution was higher in summer, 58% (95% CI 54%–62%) than spring and autumn, both 48% (95% CI 44%–52%). Winter was intermediate at 50% (95% CI 46% to 55%) and not significantly different from summer. Differences by calendar year, weekday versus weekend, and time of day were minor with overlapping confidence intervals. Female patients had higher predicted probabilities of no contribution and lower probabilities of clinical contribution than males, but these differences were not statistically significant (Fig. 5b). Complete estimates with 95% CIs are shown in Supplementary Tables 4–5.

**Fig. 6:**
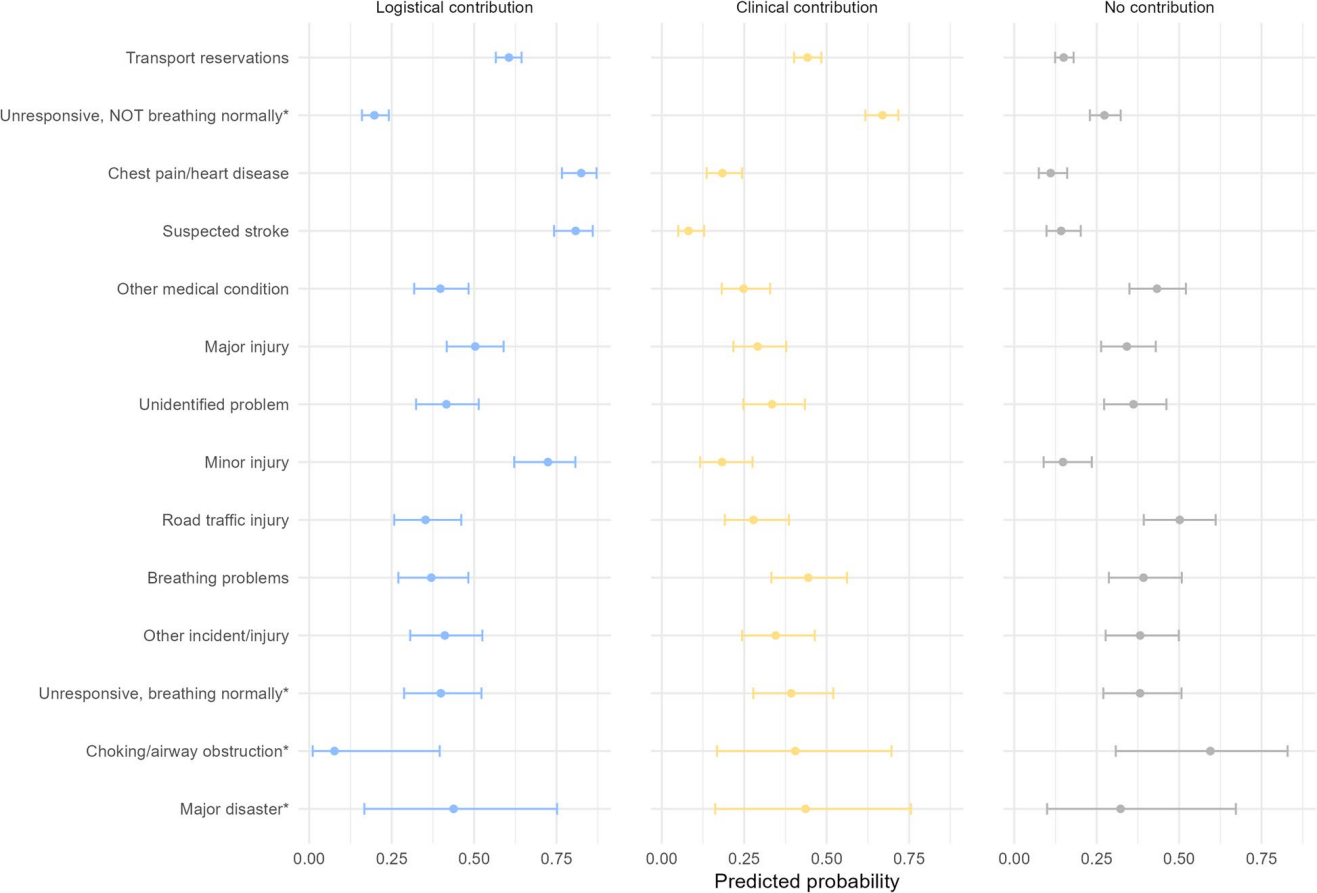
Predicted probability of contribution with 95% confidence intervals for EMCC index criteria (index groups are presented in order of decreasing frequency). Covariates: age, sex, year, season, weekday, working hours, physician, Norwegian Index for Medical Emergency Assistance (NIMN). * Red response category. EMCC – Emergency Medical Communication Centre

Incident type strongly influenced the model-based predicted probabilities of assessed contribution (Fig. 6). Logistical contribution was highest for chest pain, 82% (95% CI 77%–87%), suspected stroke, 81% (95% CI 74%–86%), minor injury, 72% (95% CI 62%–81%), and transport reservations (secondary transport), 61% (95% CI 57%–64%). Minor injury cases primarily involved patients inaccessible by road. Unresponsive patients with breathing problems had a low predicted probability of logistical contribution, 20% (95% CI 16%–24%), but the highest predicted probability of clinical contribution, 67% (95% CI 62%–72%). Road-traffic incidents had the highest predicted probability of no contribution, 50% (95% CI 39%–61%). Complete estimates with 95% CIs are provided in Supplementary Table 6.

**Fig. 7:**
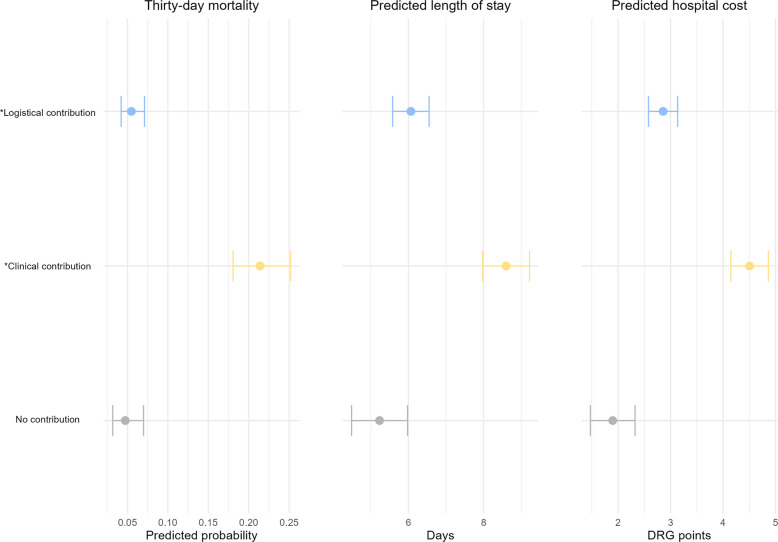
Prediction of patient outcomes with 95% confidence intervals. Covariates: age, sex, year, season, weekday, working hours, physician, logistical contribution, clinical contribution (with interaction logistical*clinical for overlapping cases). No contribution refers to the absence of both logistical and clinical contribution. Thirty-day mortality is calculated for patients with NACA < 7. Estimates for length of stay and DRG are calculated on patients with available hospital admission data from St. Olavs hospital. DRG - Diagnosis related groups, 1 DRG point = EUR 4,396/NOK 50,252 (2023, EUR 1 = NOK 11.4305), NACA- National Advisory Committee for Aeronautics

Model-based predicted outcomes by contribution category are shown in Fig. 7. Compared with logistical contribution, 30‑day mortality was higher with clinical contribution, 21% (95% CI 18%–25%) versus 5% (95% CI 4%–7%), and similar with no contribution 5% (95% CI 3%–7%). Predicted hospital length of stay was longer with clinical contribution 8.6 days (95% CI 8.0–9.2), than with logistical contribution, 6.1 days (95% CI 5.6–6.6), and no contribution, 5.2 days (95% CI 4.5–6.0). Predicted reimbursement costs were higher with clinical contribution, EUR 19,884 (95% CI 18,315–21,454), than with logistical contribution, EUR 12,578 (95% CI 11,354–13,801), and no contribution, EUR 8,288 (95% CI 6,418–10,158). Complete estimates and CIs are provided in Supplementary Table 7.

## Discussion

In our three-year dataset from Trondheim HEMS, physicians reported beneficial contribution in 74% of assessed missions. Of these, 51% had logistical contribution, 39% had clinical contribution, and 16% had both. We found no consistent temporal variation, except that logistical contribution was more common during the summer months, likely reflecting increased outdoor activity in areas inaccessible by car, like mountains and forests. Using EMCC classifications as predictors, logistical contribution was most likely in suspected incidents such as heart disease or stroke, conditions for which established guidelines emphasise rapid access to definitive treatment. Similarly, clinical contribution was more likely in incidents involving suspected critical illness, particularly in unresponsive patients with breathing problems.

A comparable pattern was observed when examining HEMS diagnoses, indicating alignment between EMCC classifications and actual clinical assessments. Logistical contribution was again associated with myocardial infarction and stroke, while clinical contribution was associated with cardiac arrest, aortic aneurysm or dissection, and intracerebral haemorrhage. We also identified convergence between clinical contribution and multiple indicators of patient severity, including NACA scores, the number of HEMS procedures performed, hospital resource use, and 30‑day mortality. These findings do not necessarily represent proven clinical benefit, but they illustrate how contribution assessments function within our setting and support convergent validity between reported contribution, established severity measures, and the underlying QI criteria. Taken together, our findings suggest that most missions were retrospectively judged as beneficial, with the logistical capacity of the helicopter emerging as the main driver of reported contribution.

The predominance of logistical contribution in our data aligns with broader system trends and changing expectations for time‑critical care. In recent years, growing awareness of time‑critical treatment for conditions such as stroke and myocardial infarction, supported by defined time‑to‑treatment targets [12, 48, 49], has driven the number of logistical missions in regions where long transport distances may jeopardise these targets. Clinical contribution was judged in almost 40% of missions, representing the most critically ill patients, as reflected by higher NACA scores, a greater number of HEMS interventions, increased hospital resource use, and higher 30‑day mortality. The decline in clinical contribution among patients classified as NACA 7 is expected, as patients declared dead at the scene derive limited benefit from either clinical or logistical contributions, even when some elements of the clinical contribution criteria may still formally apply. Most beneficial missions were either logistical or clinical, but in 16% of missions these categories overlapped, indicating combined use of both the helicopter’s logistical capacity and the crew’s clinical capabilities.

A substantial proportion of missions were assessed as having only logistical contribution, and 61% were judged as not having clinical contribution. This does not imply that the physician plays a redundant role in these missions. These assessments are retrospective and reflect only the contributions captured within the current QI framework. The contribution of an experienced anaesthetist extends beyond procedural interventions and includes diagnostic evaluation, risk assessment, decision‑making under uncertainty, and the ability to escalate or withhold treatment appropriately. Such elements of clinical judgement are not directly measured by the contribution indicators. It therefore remains unknown whether missions classified as having purely logistical contribution would have been managed equally well without a physician on board. It is also important to recognise that logistical contribution often represents the enabling step in the integrated care pathway, creating the conditions for any subsequent clinical contribution.

According to the QI guidelines, clinical contribution from HEMS may also be recorded when standard care is delivered in the absence of alternative resources, when clinical experience and decision‑making are decisive in complex situations, or when treatment is deliberately withheld on ethical grounds. In some missions, included HEMS procedures were documented without a corresponding contribution assessment, however without case review the meaning of this discrepancy is unclear. Although the contribution QIs are based on defined criteria, they ultimately rely on the attending physician’s judgement. We observed substantial variability between physicians in the proportion of missions assessed as beneficial contributions. Because each mission was evaluated only by the attending physician, inter‑rater reliability could not be estimated, and the observed variation may reflect differences in case mix, individual assessment style, and documentation practices. This raises questions about the reliability and comparability of contribution assessments across physicians and highlights the need for strengthened calibration and shared understanding of contribution criteria within the team.

The question of HEMS contribution is closely linked to triage [17, 21]. By definition, it is reasonable to infer overtriage from missions retrospectively assessed as having no contribution [50]. In our material, 26% of missions were judged as without contribution according to the criteria. Assessments of no contribution were particularly common in road traffic incidents and in cases with unclear medical conditions, where severity could be both critical and time‑sensitive. Historically, severe trauma from road traffic incidents have been a major cause of death and disability [51], and severe trauma represents one of the few clinical areas where HEMS involvement has shown causal evidence supports a survival benefit [6–8]. This has made road traffic incidents a key focus for HEMS. We also observed a higher proportion of no-contribution assessments among younger patients, possibly indicating a lower dispatch threshold in this group, consistent with reports of age‑related variation in triage performance [4, 52, 53]. Finally, RRC missions showed a higher proportion of no contribution, which may reflect case mix as well as lower dispatch thresholds and early deployment in nearby incidents [24, 54]. However, retrospective contribution assessments should not be interpreted as a reference standard for dispatch decisions, and they cannot be used to evaluate real‑time triage performance. A proportion of missions without retrospectively demonstrated contribution is expected, and to some extent necessary, given the time pressure, diagnostic uncertainty, and potential consequences of wrongful rejection. Because EMCC triage cannot perfectly identify all patients who require HEMS, deliberate overtriage has been described as a strategy to reduce undertriage in HEMS dispatch [55].

HEMS benefit may be understood as the added contribution that HEMS provides in each incident. Despite several proposals, no universal measure of HEMS benefit has been adopted [19–21]. HEMS services differ substantially because of contextual and organisational factors, and even within a single service area, operational complexity and situational variability make benefit difficult to define. Consequently, a single metric that captures benefit across all operational dimensions appears unlikely [13, 24]. Any measure of benefit should therefore avoid excessive specificity and instead combine objective baseline criteria adapted to the local operational context with structured expert judgement [56].

The EQUIPE QIs were developed and tested as part of a unified approach to quality measurement in P-EMS [24, 25]. Together, the two indicators QI 12 and QI 13 (clinical contribution and logistical contribution), offer a broad assessment of the added contribution provided by HEMS. When interpreted together, they may serve as an indirect approach to assessing HEMS benefit.

Haugland et al. proposed benchmark values for the EQUIPE indicators across Nordic HEMS services [23]. Our service met the benchmark for logistical contribution, but not for clinical contribution. Rather than indicating suboptimal dispatch, this likely reflects contextual variation in case mix, geography, and operational conditions, making universal benchmarks less meaningful for local evaluation. Benchmarks may be most appropriate for internal quality monitoring and for structured external comparison [13, 25, 54].

To advance the development of valid measures of HEMS benefit, a clearer conceptualisation of what contribution entails is needed. Existing measures have largely been operationalised and evaluated against hard outcomes, such as the provision of advanced medical procedures, deranged vital signs, severity scores, admission to critical care units, and short‑term mortality [19–21, 57]. While these remain essential, they capture only part of the potential contribution of HEMS. Other patient‑centred and system‑level outcomes, such as long‑term recovery, functional status, health‑related quality of life, return to work, sustained independence, and avoidance of complications, may be equally important when considering the broader impact of HEMS [12, 16]. A more comprehensive understanding of contribution, combined with improved calibration among physicians, may enhance the validity and consistency of future work.

### Strengths and limitations

Contribution assessments were based on retrospective judgements by attending physicians, introducing the possibility of retrospective bias, even with explicit guidelines, as evaluators may be influenced by known outcomes or by their own involvement [4, 25, 58]. We observed substantial variation between physicians in the proportion of missions assessed as beneficial contributions. Because each mission was evaluated only by the attending physician, inter‑rater reliability could not be estimated. The observed variation may reflect differences in case mix, individual assessment style, or documentation practices. This raises questions about the reliability and comparability of contribution assessments across physicians and highlights the need for strengthened calibration and a shared understanding of the contribution criteria within the team. These uncertainties should be kept in mind when interpreting the reported results.

Patient outcome measures were limited and captured only indirect aspects of potential clinical contribution [4, 12]. We lacked counterfactual data to estimate patient trajectories without HEMS involvement [22, 52, 59], meaning that our findings cannot be interpreted as causal evidence of patient‑level impact [4, 22]. Moreover, the contribution QIs capture only certain dimensions of HEMS activity and do not fully reflect broader contributions such as risk assessment and decision-making under uncertainty.

Despite these limitations, the study has several strengths. The dataset encompasses three consecutive years of uninterrupted operations, with near‑complete contribution assessments. The integration of EMCC, HEMS, and hospital data provides a comprehensive overview of both operational and clinical characteristics, enabling robust descriptive analysis. The use of standardised QI criteria further supports consistency in reporting and facilitates comparison with other services.

## Conclusion

Across three years of retrospective physician‑reported assessments, three quarters of missions in the Trondheim HEMS were judged as beneficial contributions. About half of the missions were considered to involve a contribution from helicopter capabilities, and 39 percent were judged to provide clinical contribution from the crew, with a 16 percent overlap. About one quarter of HEMS missions were assessed as having no contribution relative to ordinary EMS. The observed level of no contribution is broadly consistent with a service operating within an expected range, where a degree of no contribution is anticipated to avoid undertriage. It likely reflects contextual uncertainty at the time of dispatch and the inherent limitations of retrospective assessment rather than unavoidable overtriage.

Although assessments were based on structured criteria developed by the EQUIPE group, we observed substantial variation between physicians. This variation likely reflects differences in case mix, assessment style, and interpretation of the contribution indicators, underscoring the need for improved calibration and a shared understanding before such assessments are used for causal conclusions. A shared understanding of the framework for HEMS contribution, combined with more consistent application of the criteria, may improve the validity, reliability, and future utility of these assessments.

## Supplementary Information

Supplementary Material 1.

## Data Availability

SQL code for extracting and merging data from HMN Datavarehus, as well as R-code for statistical analyses, and for generating tables and figures, are available on GitHub: [https://github.com/larsnaess/HEMS-contribution]. The data supporting the findings of this study are not publicly available due to personal data and information security regulations. However, data may be made available upon reasonable request to the Central Norway Regional Health Authority. Contact: Central Norway Regional Health Authority. P.O. Box 464. N-7501 Stjørdal, Norway. Email: postmottak@helse-midt.no. Before requesting data, ethical approval must be obtained from the Regional Committee for Medical and Health Research Ethics (REK Midt). Applications can be submitted via https://rekportalen.no/en/, and inquiries directed to rek-midt@mh.ntnu.no.

## Abbreviations

CI: Confidence Interval
CPR: Cardiopulmonary Resuscitation
CVC: Central Venous Catheter
DRG: Diagnosis-Related Group
ECMO: Extracorporeal Membrane Oxygenation
EHR: Electronic Health Record
EMCC: Emergency Medical Communication Centre
EMS: Emergency Medical Services
ePCR: Electronic Patient Care Report
EQUIPE: Establishing Quality Indicators in P-EMS
EUR: Euro (currency of the eurozone)
HEMS: Helicopter Emergency Medical Services
GEMS: Ground Emergency Medical Services
ICD-10: International Classification of Diseases, 10th Revision
IT: Information Technology
NACA: National Advisory Committee for Aeronautics
NOK: Norwegian Krone (currency of Norway)
PID: Personal Identifier
P-EMS: Physician-staffed Emergency Medical Services
QI: Quality Indicator
RRC: Rapid Response Car
SAR: Search And Rescue
SQL: Structured Query Language
STEMI: ST-Elevation Myocardial Infarction
