# Supplementary material for: Assessment of clinical and logistical contribution in a Norwegian helicopter emergency medical service using integrated data: a retrospective observational study
Source: Scand J Trauma Resusc Emerg Med. 2026 Apr 17;34:93. doi: 10.1186/s13049-026-01615-3 (PMC13214417; doi:10.1186/s13049-026-01615-3)
Supplement: Supplementary file 1 — Supplementary Material 1. [file 13049_2026_1615_MOESM1_ESM.pdf]

Assessment of clinical and logistical contribution in a Norwegian Helicopter Emergency Medical Service using integrated data: A retrospective observational study

Supplementary files

Content

Supplementary Figure 1 (emergency care pathway and data sources) 2

Supplementary Figure 2 (subcategories for contribution assessments) 3

Supplementary Figure 3 (screenshot from QI 12: assessment of clinical contribution in QI application - Norwegian)... 4

Supplementary Figure 4 (screenshot from QI 13: assessment of logistical contribution in QI application - Norwegian)... 5

Supplementary Table 1 (description of NACA score)... 6

Supplementary Table 2 (associations with contribution assessments – Figure 3-4)........ 7

Supplementary Table 3 (HEMS diagnosis and contribution assessment)....................... 8

Supplementary Table 4 (contribution assessment per age – Figure 5a) 9

Supplementary Table 5 (contribution assessment for time factors and sex – Figure 5b) 11

Supplementary Table 6 (contribution assessment for incident types – Figure 6)........... 12

Supplementary Table 7 (predicted outcomes based on contribution assessment – Figure 7) 13

List of abbreviations 14

Supplementary Figure 1 (emergency care pathway and data sources)

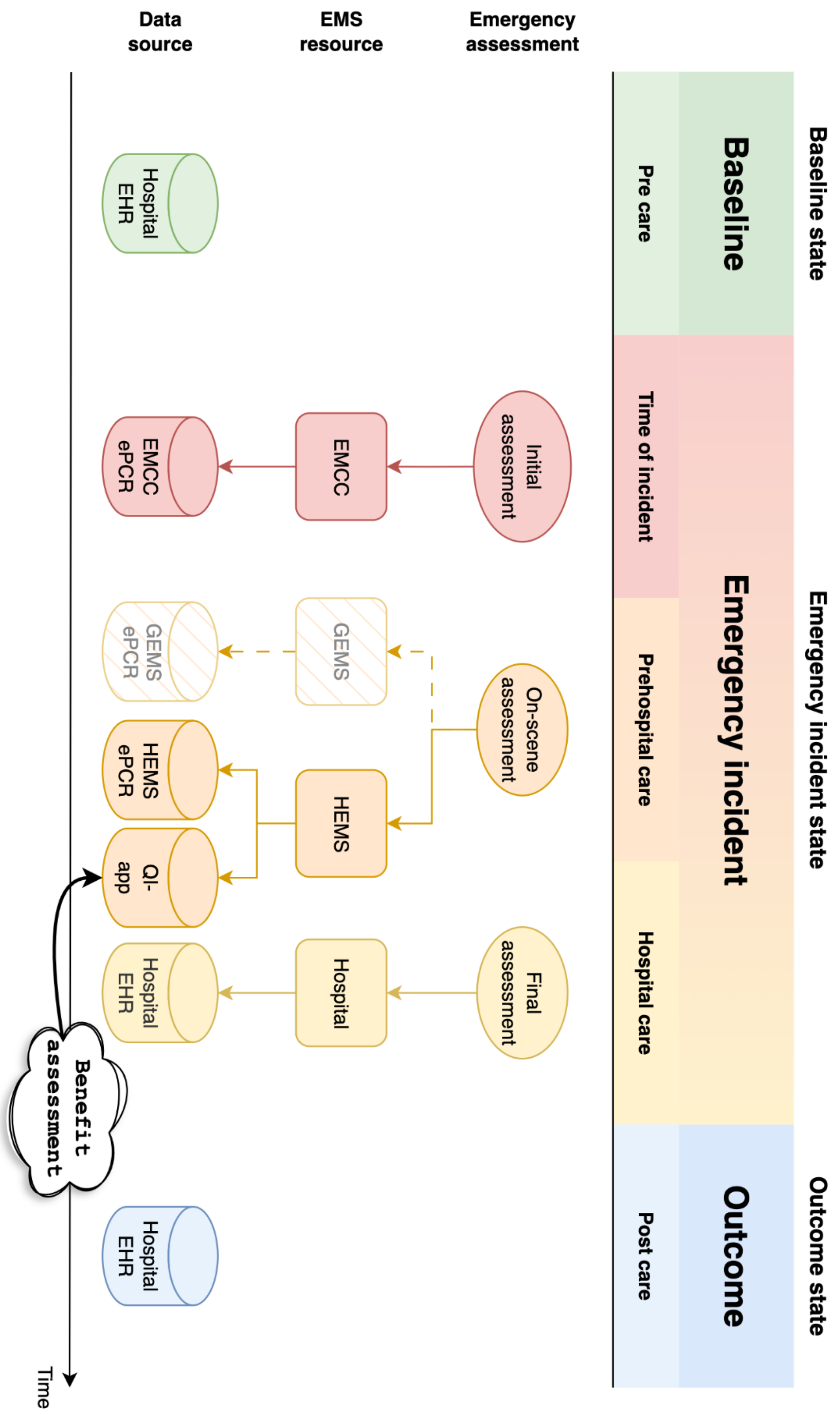

Supplementary Figure 1: Overview of the emergency care pathway, EMS resource involvement and data flow from the emergency call to hospital discharge.

EHR – Electronic Health Record, EMCC – Emergency Medical Communication Centre, EMS – Emergency Medical Services, ePCR – electronic Patient Care Report, GEMS – Ground Emergency Medical Services, HEMS – Helicopter Emergency Medical Services, QI-app – Quality Indicator application.

Supplementary Figure 2 (subcategories for contribution assessments)

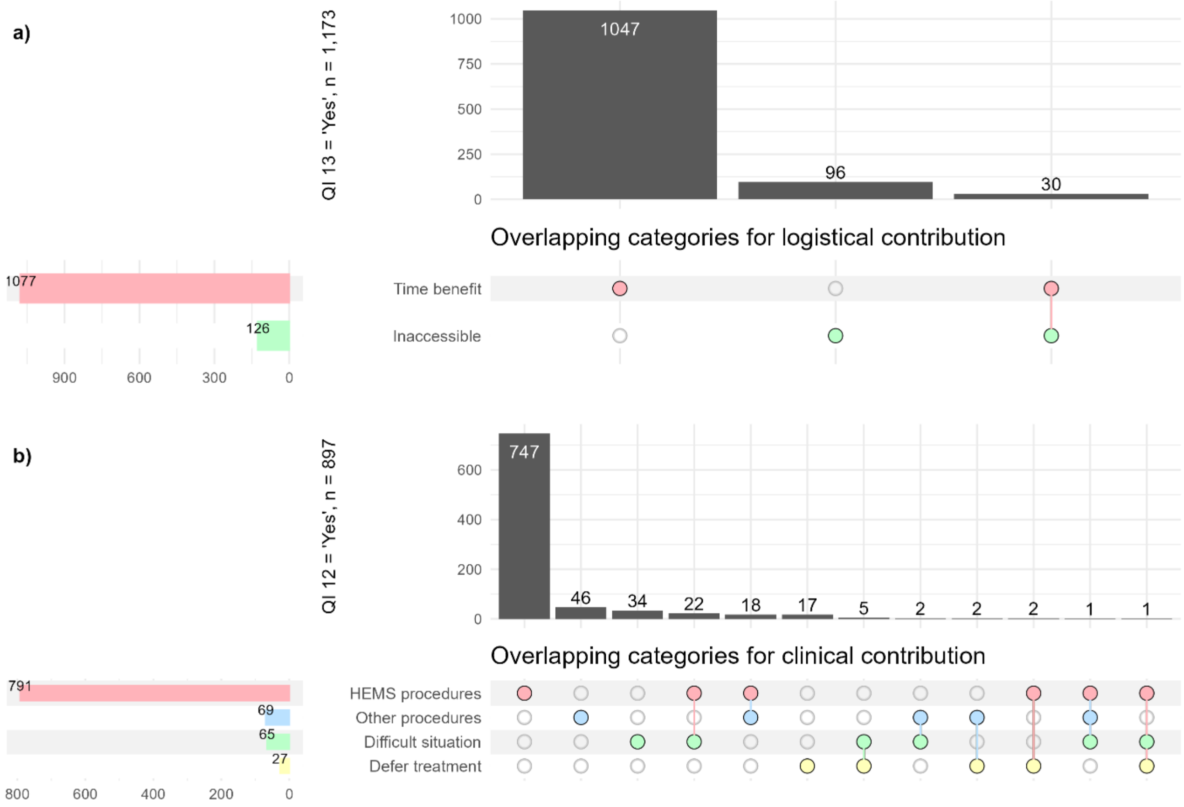

Supplementary Figure 2: Detailed description of the distribution of subcategories for a) logistical contribution and b) clinical contribution. HEMS – Helicopter Emergency Medical Services

Supplementary Figure 3 (screenshot from QI 12: assessment of clinical contribution in QI application - Norwegian)

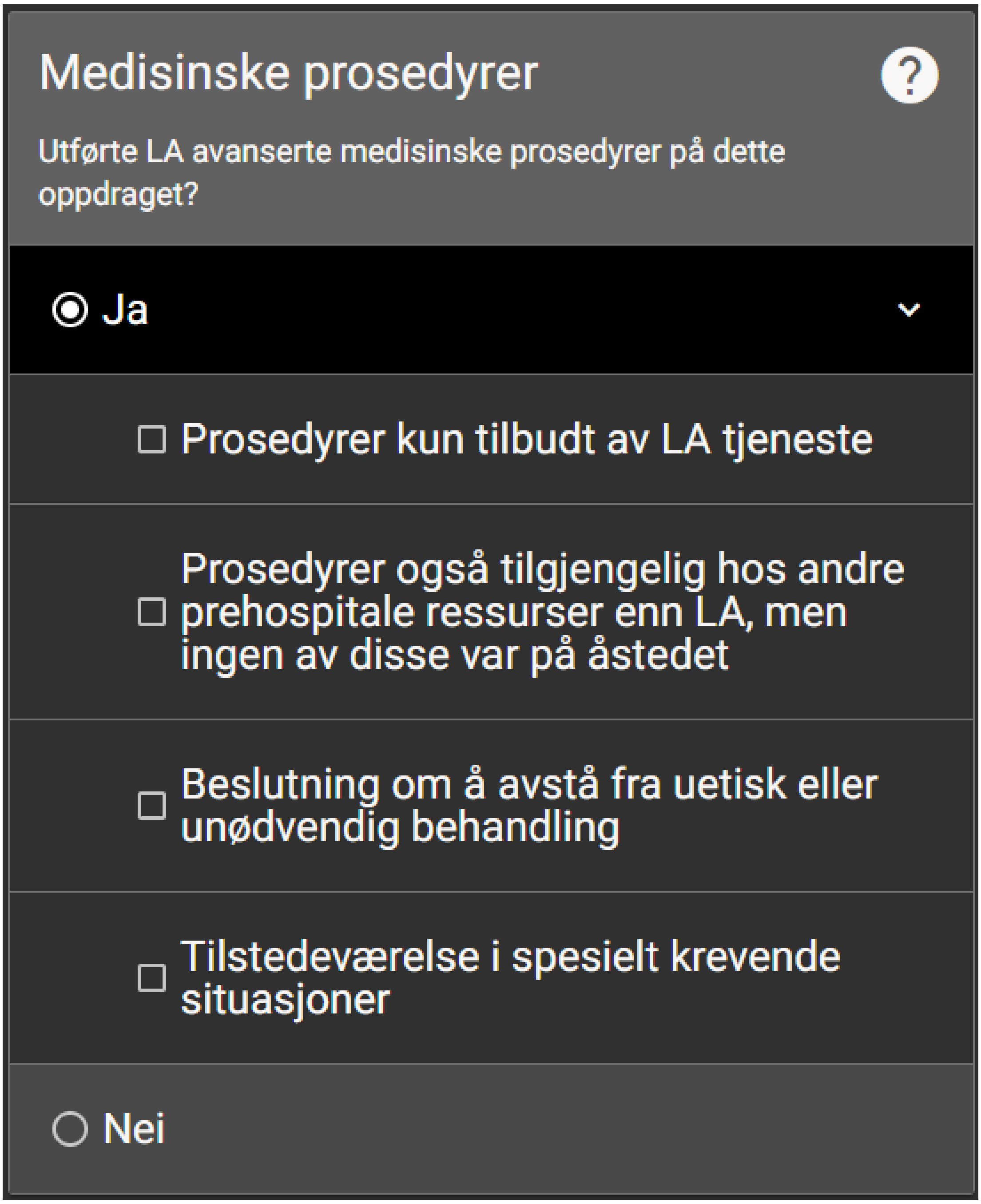

Supplementary Figure 4 (screenshot from QI 13: assessment of logistical contribution in QI application - Norwegian)

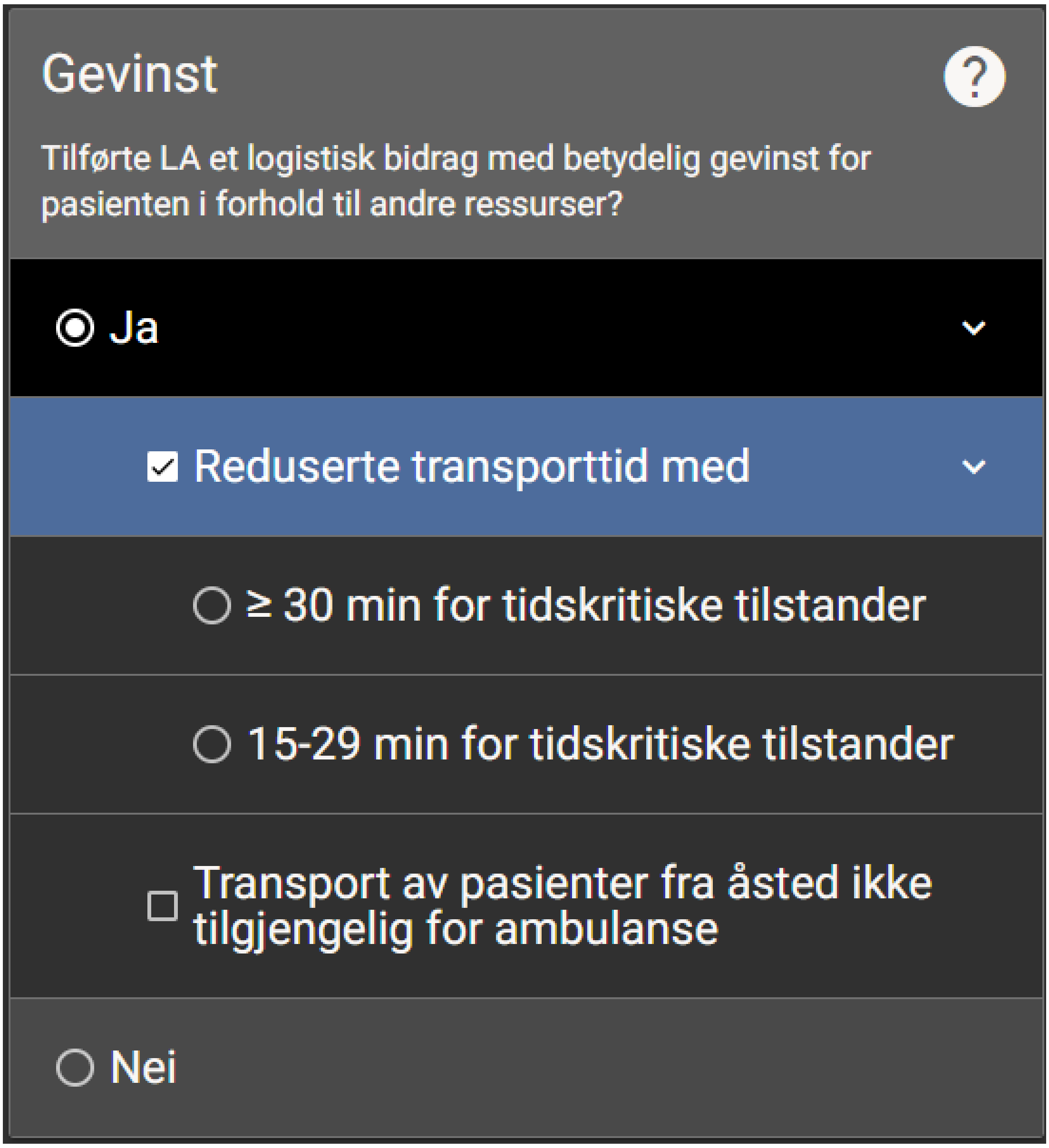

Supplementary Table 1 (description of NACA score)

Supplementary Table 1: NACA score

Score	Patient state
NACA 0	No injury or disease
NACA 1	Injuries/diseases without any need for acute physician's care
NACA 2	Injuries/diseases requiring examination and therapy by a physician, but hospital admission is not indicated
NACA 3	Injuries/diseases without acute threat to life but requiring hospital admission
NACA 4	Injuries/diseases that can possibly lead to deterioration of vital signs
NACA 5	Injuries/diseases with acute threat to life
NACA 6	Injuries/diseases transported after successful resuscitation of vital signs
NACA 7	Lethal injuries or diseases (with or without resuscitation attempts)

NACA - National Advisory Committee for Aeronautics

Supplementary Table 2 (associations with contribution assessments – Figure 3-4)

Supplementary Table 2: HEMS characteristics with corresponding contribution assessments

Contribution assessment	Logistical contribution* (row %)	Clinical contribution* (row %)	Double contribution* (row %)	No contribution (row %)	Total
Overall	1173 (51)	897 (39)	374 (16)	590 (26)	2286
Mission type					
Primary mission	914 (50)	642 (35)	251 (14)	535 (29)	1840
Secondary mission	255 (59)	248 (57)	121 (28)	52 (12)	434
SAR / Other	<5 (-)	7 (58)	<5 (-)	<5 (-)	12
Vessel					
Helicopter	1172 (69)	602 (36)	373 (22)	290 (17)	1691
RRC	<5 (-)	270 (48)	<5 (-)	290 (52)	560
Other	<5 (-)	25 (71)	<5 (-)	10 (29)	35
NACA score†					
NACA 1-3	232 (48)	53 (11)	22 (5)	224 (46)	487
NACA 4	587 (68)	172 (20)	98 (11)	198 (23)	859
NACA 5	250 (55)	300 (66)	162 (36)	68 (15)	456
NACA 6	100 (40)	206 (83)	90 (36)	32 (13)	248
NACA 7	<5 (-)	165 (71)	<5 (-)	68 (29)	234
Number of advanced procedures					
0 procedures	715 (58)	160 (13)	82 (7)	449 (36)	1242
1 procedure	233 (57)	182 (44)	94 (23)	90 (22)	411
2 procedures	76 (39)	136 (70)	52 (27)	34 (18)	194
3-4 procedures	79 (27)	275 (94)	77 (26)	16 (5)	293
5 or more procedures	70 (48)	144 (99)	69 (47)	<5 (-)	146

**Overlapping categories, HEMS – Helicopter Emergency Medical Services, RRC – Rapid Response Car, SAR – Search and Rescue. †Unknown NACA n = 2*

Supplementary Table 3 (HEMS diagnosis and contribution assessment)

Supplementary Table 3: Most frequent truncated HEMS diagnosis codes (ICD-10 3-character level) with corresponding contribution assessments

Contribution assessment	Logistical contribution* (row %)	Clinical contribution* (row %)	Double contribution* (row %)	No contribution (row %)	Total
Overall	1173 (51)	897 (39)	374 (16)	590 (26)	2286
HEMS diagnosis (ICD10)					
I46 - Cardiac arrest	66 (17)	296 (77)	57 (15)	78 (20)	383
I21 - Acute myocardial infarction	204 (95)	37 (17)	33 (15)	7 (3)	215
I64 - Stroke, not specified as haemorrhage or infarction	124 (87)	17 (12)	14 (10)	16 (11)	143
R56 - Convulsions	19 (21)	20 (22)	6 (7)	58 (64)	91
J96 - Respiratory failure	23 (28)	48 (58)	17 (20)	29 (35)	83
S06 - Intracranial injury	40 (55)	18 (25)	16 (22)	31 (42)	73
I61 - Intracerebral haemorrhage	33 (62)	33 (62)	18 (34)	5 (9)	53
R55 - Syncope and collapse	9 (22)	5 (12)	<5 (-)	28 (68)	41
I63 - Cerebral infarction	33 (87)	<5 (-)	<5 (-)	<5 (-)	38
I71 - Aortic aneurysm and dissection	25 (69)	26 (72)	18 (50)	<5 (-)	36
I22 - Subsequent myocardial infarction	32 (97)	10 (30)	9 (27)	<5 (-)	33
R10 - Abdominal and pelvic pain	17 (55)	<5 (-)	<5 (-)	13 (42)	31
A41 - Other sepsis	15 (54)	11 (39)	5 (18)	7 (25)	28
S22 - Fracture of rib(s)	15 (56)	10 (37)	7 (26)	9 (33)	27
S72 - Fracture of femur	13 (50)	11 (42)	<5 (-)	6 (23)	26
S09 - Other and unspecified injuries of head	15 (60)	10 (40)	5 (20)	5 (20)	25
R40 - Somnolence, stupor and coma	8 (38)	6 (29)	<5 (-)	9 (43)	21
S82 - Fracture of lower leg, including ankle	11 (52)	5 (24)	<5 (-)	6 (29)	21
Other (<20 per diagnosis)	471 (51)	327 (36)	157 (17)	275 (30)	916
NA	<5 (-)	<5 (-)	<5 (-)	<5 (-)	<5 (-)

**Overlapping categories, HEMS – Helicopter Emergency Medical Services, ICD-10 - International Classification of Diseases, 10th Revision*

Supplementary Table 4 (contribution assessment per age – Figure 5a)

Supplementary Table 4: Predicted probabilities for contribution assessment based on patient age

Patient age	Cases	Logistical contribution		Clinical contribution		No contribution	
(Years)	(n=)	Estimate	95% CI	Estimate	95% CI	Estimate	95% CI
0	89	0,40	(0,31-0,50)	0,54	(0,44-0,64)	0,24	(0,17-0,34)
2	28	0,40	(0,32-0,50)	0,53	(0,44-0,63)	0,25	(0,18-0,34)
4	15	0,41	(0,32-0,50)	0,52	(0,43-0,62)	0,25	(0,18-0,34)
6	12	0,41	(0,33-0,50)	0,52	(0,43-0,60)	0,26	(0,19-0,35)
8	10	0,42	(0,34-0,50)	0,51	(0,42-0,59)	0,26	(0,19-0,35)
10	8	0,42	(0,34-0,50)	0,50	(0,41-0,58)	0,27	(0,20-0,35)
12	10	0,42	(0,35-0,51)	0,49	(0,40-0,58)	0,27	(0,20-0,36)
14	11	0,43	(0,35-0,51)	0,48	(0,40-0,57)	0,27	(0,20-0,36)
16	6	0,43	(0,35-0,52)	0,48	(0,39-0,56)	0,28	(0,20-0,36)
18	19	0,44	(0,36-0,53)	0,47	(0,38-0,56)	0,28	(0,20-0,37)
20	10	0,45	(0,36-0,53)	0,46	(0,38-0,56)	0,28	(0,20-0,37)
22	14	0,45	(0,37-0,54)	0,46	(0,37-0,55)	0,28	(0,20-0,37)
24	13	0,46	(0,37-0,55)	0,46	(0,37-0,55)	0,27	(0,20-0,37)
26	16	0,47	(0,38-0,56)	0,46	(0,37-0,55)	0,27	(0,19-0,36)
28	14	0,48	(0,39-0,57)	0,46	(0,37-0,55)	0,26	(0,19-0,36)
30	13	0,49	(0,40-0,58)	0,46	(0,37-0,55)	0,26	(0,18-0,35)
32	14	0,50	(0,41-0,59)	0,46	(0,37-0,56)	0,25	(0,18-0,34)
34	18	0,51	(0,42-0,60)	0,47	(0,38-0,56)	0,24	(0,17-0,33)
36	<5	0,52	(0,44-0,61)	0,47	(0,38-0,57)	0,23	(0,16-0,31)
38	8	0,54	(0,45-0,62)	0,48	(0,39-0,57)	0,21	(0,15-0,29)
40	14	0,55	(0,47-0,63)	0,49	(0,41-0,58)	0,20	(0,14-0,28)
42	14	0,56	(0,48-0,64)	0,50	(0,42-0,59)	0,19	(0,13-0,26)
44	18	0,58	(0,50-0,66)	0,52	(0,43-0,60)	0,17	(0,12-0,24)
46	18	0,59	(0,51-0,67)	0,53	(0,44-0,61)	0,16	(0,11-0,22)
48	23	0,60	(0,52-0,68)	0,54	(0,46-0,63)	0,15	(0,10-0,21)
50	26	0,61	(0,53-0,69)	0,56	(0,47-0,64)	0,14	(0,10-0,20)
52	21	0,62	(0,54-0,70)	0,57	(0,49-0,65)	0,13	(0,09-0,18)
54	26	0,63	(0,55-0,70)	0,58	(0,50-0,66)	0,12	(0,08-0,17)
56	39	0,64	(0,56-0,71)	0,60	(0,51-0,68)	0,11	(0,08-0,16)
58	47	0,64	(0,56-0,71)	0,61	(0,52-0,69)	0,11	(0,07-0,15)
60	33	0,64	(0,56-0,71)	0,62	(0,54-0,69)	0,10	(0,07-0,15)
62	40	0,63	(0,56-0,71)	0,63	(0,55-0,70)	0,10	(0,07-0,14)
64	32	0,63	(0,55-0,70)	0,63	(0,56-0,71)	0,09	(0,07-0,14)
66	48	0,62	(0,54-0,69)	0,64	(0,56-0,71)	0,09	(0,06-0,13)
68	40	0,61	(0,53-0,68)	0,64	(0,57-0,71)	0,09	(0,06-0,13)
70	54	0,60	(0,52-0,68)	0,65	(0,57-0,72)	0,09	(0,06-0,14)
72	41	0,59	(0,52-0,67)	0,65	(0,57-0,72)	0,10	(0,07-0,14)
74	68	0,59	(0,51-0,66)	0,64	(0,56-0,72)	0,10	(0,07-0,14)
76	62	0,59	(0,51-0,66)	0,64	(0,56-0,71)	0,10	(0,07-0,14)

Patient age	Cases	Logistical contribution		Clinical contribution		No contribution	
(Years)	(n=)	Estimate	95% CI	Estimate	95% CI	Estimate	95% CI
78	50	0,59	(0,51-0,66)	0,63	(0,55-0,71)	0,10	(0,07-0,15)
80	34	0,59	(0,51-0,67)	0,62	(0,54-0,70)	0,11	(0,07-0,15)
82	24	0,60	(0,51-0,67)	0,61	(0,53-0,69)	0,11	(0,07-0,16)
84	24	0,60	(0,52-0,69)	0,60	(0,51-0,68)	0,12	(0,08-0,17)
86	13	0,61	(0,52-0,70)	0,58	(0,48-0,68)	0,12	(0,08-0,18)
88	12	0,63	(0,51-0,73)	0,57	(0,45-0,68)	0,13	(0,08-0,20)
90	<5	0,64	(0,51-0,75)	0,55	(0,41-0,68)	0,13	(0,07-0,22)
92	<5	0,65	(0,50-0,77)	0,53	(0,37-0,68)	0,14	(0,07-0,25)
94	<5	0,66	(0,50-0,80)	0,51	(0,33-0,69)	0,14	(0,07-0,28)
96	<5	0,68	(0,49-0,82)	0,49	(0,30-0,69)	0,15	(0,06-0,32)
98	<5	0,69	(0,48-0,85)	0,47	(0,26-0,70)	0,16	(0,06-0,36)
100	<5	0,71	(0,47-0,87)	0,45	(0,22-0,70)	0,17	(0,06-0,40)

Covariates: age, sex, year, season, weekday, working hours, physician

CI - Confidence Interval

Supplementary Table 5 (contribution assessment for time factors and sex – Figure 5b)

Supplementary Table 5: Predicted probabilities for contribution assessment based on time factors and patient sex

Predictor	Cases	Logistical contribution		Clinical contribution		No contribution	
Variable	n	Estimate	95% CI	Estimate	95% CI	Estimate	95% CI
Year							
2022	807	0,53	(0,49-0,56)	0,40	(0,36-0,43)	0,23	(0,20-0,26)
2023	763	0,53	(0,49-0,57)	0,37	(0,34-0,41)	0,23	(0,20-0,26)
2024	716	0,48	(0,44-0,51)	0,37	(0,34-0,41)	0,26	(0,23-0,30)
Season							
Winter	517	0,50	(0,46-0,55)	0,36	(0,32-0,41)	0,25	(0,21-0,29)
Spring	621	0,48	(0,44-0,52)	0,39	(0,35-0,43)	0,27	(0,23-0,31)
Summer	607	0,58	(0,54-0,62)	0,36	(0,32-0,40)	0,21	(0,18-0,24)
Autumn	541	0,48	(0,44-0,52)	0,41	(0,37-0,46)	0,23	(0,20-0,27)
Time of week							
Weekday	1444	0,52	(0,49-0,54)	0,38	(0,36-0,41)	0,23	(0,21-0,26)
Weekend	842	0,50	(0,47-0,54)	0,37	(0,34-0,41)	0,25	(0,22-0,28)
Shift							
0000-0600	271	0,49	(0,43-0,55)	0,42	(0,36-0,48)	0,24	(0,19-0,29)
0600-1200	582	0,54	(0,50-0,58)	0,35	(0,31-0,39)	0,22	(0,19-0,26)
1200-1800	829	0,52	(0,49-0,56)	0,37	(0,33-0,40)	0,23	(0,21-0,27)
1800-2400	604	0,48	(0,44-0,52)	0,41	(0,37-0,45)	0,26	(0,23-0,30)
Sex							
Female	816	0,49	(0,46-0,53)	0,34	(0,31-0,38)	0,27	(0,24-0,31)
Male	1470	0,53	(0,50-0,55)	0,40	(0,38-0,43)	0,22	(0,20-0,24)

Covariates: age, sex, year, season, weekday, working hours, physician

CI - Confidence Interval

Supplementary Table 6 (contribution assessment for incident types – Figure 6)

Supplementary Table 6: Predicted probability of contribution for Norwegian Index for Medical Emergency Assistance

Predictor	Cases	Logistical contribution		Clinical contribution		No contribution	
Variable	n	Estimate	95% CI	Estimate	95% CI	Estimate	95% CI
Transport reservations	636	0,61	(0,57-0,64)	0,44	(0,40-0,48)	0,15	(0,12-0,18)
Unresponsive, NOT breathing normally*	368	0,20	(0,16-0,24)	0,67	(0,62-0,72)	0,27	(0,23-0,32)
Chest pain/heart disease	202	0,82	(0,77-0,87)	0,18	(0,14-0,24)	0,11	(0,07-0,16)
Suspected stroke	174	0,81	(0,74-0,86)	0,08	(0,05-0,13)	0,14	(0,10-0,20)
Other medical condition	141	0,40	(0,32-0,48)	0,25	(0,18-0,33)	0,43	(0,35-0,52)
Major injury	133	0,50	(0,42-0,59)	0,29	(0,22-0,38)	0,34	(0,26-0,43)
Unidentified problem	105	0,42	(0,32-0,51)	0,33	(0,25-0,43)	0,36	(0,27-0,46)
Minor injury	91	0,72	(0,62-0,81)	0,18	(0,12-0,28)	0,15	(0,09-0,23)
Road traffic injury	86	0,35	(0,26-0,46)	0,28	(0,19-0,39)	0,50	(0,39-0,61)
Breathing problems	80	0,37	(0,27-0,48)	0,44	(0,33-0,56)	0,39	(0,29-0,51)
Other incident/injury	79	0,41	(0,31-0,52)	0,35	(0,24-0,46)	0,38	(0,28-0,50)
Unresponsive, breathing normally*	67	0,40	(0,29-0,52)	0,39	(0,28-0,52)	0,38	(0,27-0,51)
Choking/airway obstruction*	12	0,08	(0,01-0,40)	0,40	(0,17-0,70)	0,59	(0,31-0,83)
Major disaster*	9	0,44	(0,17-0,75)	0,44	(0,16-0,76)	0,32	(0,10-0,67)

Covariates: age, sex, year, season, weekday, working hours, physician, Norwegian Index for Medical Emergency Assistance

** Emergency Medical Communication Centre red response category*

CI - Confidence Interval

Supplementary Table 7 (predicted outcomes based on contribution assessment – Figure 7)

Supplementary Table 7: Prediction of patient outcomes based on assessment of contribution

Predictor	30-day mortality			Length of stay			Hospital costs		
Contribution type	*n*	*Probability*	*95% CI*	*n*	*Days*	*95% CI*	*n*	*DRG*	*95% CI*
Logistical	1111	0,05	(0,04-0,07)	1033	6,1	(5,6-6,6)	1033	2,9	(2,6-3,1)
Clinical	689	0,21	(0,18-0,25)	648	8,6	(8,0-9,2)	648	4,5	(4,1-4,9)
No	500	0,05	(0,03-0,07)	452	5,2	(4,5-6,0)	452	1,9	(1,5-2,3)

*Covariates: logistic contribution, clinical contribution, age, sex, year, season, weekday, working hours, physician (with interaction logistic*clinical for overlapping cases).*

CI - Confidence Interval

DRG - Diagnosis Related Groups, 1 DRG-point = NOK 50 252 / EUR 4 396 (2023)

List of abbreviations

CI	Confidence Interval
CPR	Cardiopulmonary Resuscitation
CVC	Central Venous Catheter
DRG	Diagnosis-Related Group
ECMO	Extracorporeal Membrane Oxygenation
EHR	Electronic Health Record
EMCC	Emergency Medical Communication Centre
EMS	Emergency Medical Services
ePCR	electronic Patient Care Report
EQUIPE	Establishing Quality Indicators in P-EMS
EUR	Euro (currency of the eurozone)
HEMS	Helicopter Emergency Medical Services
GEMS	Ground Emergency Medical Services
ICD-10	International Classification of Diseases, 10th Revision
IT	Information Technology
NACA	National Advisory Committee for Aeronautics
NOK	Norwegian Krone (currency of Norway)
PID	Personal Identifier
P-EMS	Physician-staffed Emergency Medical Services
QI	Quality Indicator
RRC	Rapid Response Car
SAR	Search And Rescue
SQL	Structured Query Language
STEMI	ST-Elevation Myocardial Infarction
